# Aptamer-associated colorimetric reverse transcription loop-mediated isothermal amplification assay for detection of dengue virus

**DOI:** 10.1128/spectrum.03583-23

**Published:** 2024-07-24

**Authors:** Vitória de Oliveira Arruda, Luiz Ricardo Goulart Filho, Adriana Freitas Neves

**Affiliations:** 1 Institute of Physics, Universidade Federal de Catalão, Catalão, Goiás, Brazil; 2 Institute of Biotechnology, Universidade Federal de Uberlândia, Uberlândia, Minas Gerais, Brazil; 3 Institute of Biotechnology, Universidade Federal de Catalão, Catalão, Goiás, Brazil; National Chung Hsing University, Taichung, Taiwan

**Keywords:** molecular biology, arboviruses, diagnosis, point-of-care

## Abstract

Current diagnostic methods for dengue, such as serological tests, have limitations in terms of cross-reactivity with other viruses. To address this issue, we explored the potential of combining the loop-mediated isothermal amplification (LAMP) technique with the affinity of aptamers to develop point-of-care testing. In this study, we utilized 60 serum samples. An aptamer capable of binding to the dengue virus was employed as a platform for capturing genetic material, and its performance was compared to a commercial kit. Dengue virus was detected through RT-PCR and colorimetric reverse transcription loop-mediated isothermal amplification (RT-LAMP), allowing visual observation of the results without the need for equipment. In the context of the aptamer LAMP assay, our analysis revealed the detection of the dengue virus in 38 out of 60 samples, with 95% sensitivity and 100% specificity compared to RT-PCR and/or APTA-RT-PCR. Importantly, we observed no cross-reaction when assessing samples positive for the zika virus, underscoring the assay’s selectivity. This innovative aptameric capture of the viral RNA in combination with the RT-LAMP (APTA-RT-LAMP) method has the potential to offer valuable molecular insights into neglected infectious diseases in a simpler and faster manner.

**IMPORTANCE:**

Dengue is a neglected tropical disease of significant epidemiological importance in tropical and subtropical countries. Current diagnostics for this infection present challenges, such as cross-reactivity in serological tests. Finding ways to enhance the diagnosis of this disease is crucial, given the absence of specific treatments. An accurate, simple, and effective diagnosis contributes to the improved management of infected individuals. In this context, our work combines molecular biology techniques, such as isothermal loop amplification, with aptamers to detect the dengue virus in biological samples. Our method produces colorimetric results based on a color change, with outcomes available in less than 2 hours. Moreover, it requires simpler equipment compared to molecular PCR tests.

## INTRODUCTION

Dengue is a disease caused by an arbovirus, which is a virus transmitted by arthropods. The dengue virus (DENV) belongs to the Flaviviridae family and the Flavivirus genus, and it has four serotypes (DENV-1, DENV-2, DENV-3, and DENV-4). Transmission occurs after being bitten by a female mosquito of the *Aedes* genus. It is estimated that approximately 3 billion people live in areas conducive to the growth of the vector, namely tropical and subtropical regions (1, 2).

Dengue virus infection can manifest in a wide range of symptoms, including high fever, headache, malaise, joint pain, photophobia, retro-orbital pain, lack of appetite, abdominal pain, nausea, diarrhea, and loss of appetite and weight. Clinical findings may include leukopenia and thrombocytopenia, and severe cases of dengue infection can lead to severe bleeding (3).

Diagnostic platforms for dengue are based on the duration of symptoms. Within the first 5 days, the most recommended techniques are virus isolation, detection of viral DNA through polymerase chain reaction (PCR), or detection of serum antigens such as NS1. After this period, antibody detection using serological methods, particularly IgM or IgG reactivity, is more appropriate (4).

However, serological methods may cross-react with other arboviruses such as West Nile virus (WNV), yellow fever virus (YFV), zika virus (ZIKV), Japanese encephalitis virus (JEV), chikungunya virus (CHIKV), and mayaro virus (MAYV) (5, 6). Another challenge is the wide variation in screening tests among commercial kits, with sensitivity ranging from 56% to 92% and specificity from 64% to 97% (7).

The detection of NS1 protein differs between serotypes, and in cases of secondary infections, this method has reduced sensitivity (8, 9). Molecular biology assays like PCR provide more sensitive and specific results, but they require specialized personnel and equipment in a laboratory setting, which may not be readily available in remote or resource-poor areas where dengue is endemic (10).

In 1998, the Japanese company Eiken Chemical Co. Ltd. developed a molecular method for nucleic acid amplification called loop-mediated isothermal amplification (LAMP) (11). This technique is precise and can increase the amount of amplified DNA by up to a billion copies in less than an hour. Isothermal amplification can be performed using basic equipment, such as a dry or water bath. One key difference between LAMP and PCR is the number of primers used per reaction, with LAMP employing four to six primers, enabling the distinction of up to eight specific sites in the DNA template. The final product of LAMP is multiple DNA fragments (11, 12). Various visualization methods can be used for LAMP results, such as agarose gel electrophoresis, real-time fluorescence colorimetric detection using fluorescent dyes, and turbidity (13–15).

In addition, oligonucleotide aptamers generated through a process called Systematic Evolution of Ligands by Exponential Enrichment (SELEX) have potential applications in dengue disease (16). Oligonucleotide aptamers are single-stranded DNA or RNA molecules that bind to a target with high affinity and specificity (17, 18). Due to their non-immunogenic and non-toxic nature, aptamers are promising candidates for therapeutic applications (19).

Aptamers also offer advantages in diagnosis, such as being coupled with fluorophores and/or nanoparticles to create biosensors (20). Furthermore, aptamers can be used in sample processing to extract genetic material due to their specific binding properties (21).

In this study, we present an association of aptamers aimed at capturing the dengue virus in biological samples and detecting it using the LAMP technique. The colorimetric results, visible to the naked eye in around one hour, indicate the potential of our new methodology called APTA-RT-LAMP for DENV detection which can be used in the field as a point-of-care test. Our APTA-RT-LAMP, based on the Nucleic Acid Amplification Test (NAAT), is very cost-effective compared to PCR, which utilizes conventional RNA extraction methods. Its accessibility, even in regions lacking infrastructure, creates opportunities for the detection of other arboviruses and neglected diseases. Ultimately, this could significantly improve epidemiological control and treatment if made widely available.

## MATERIALS AND METHODS

### Material and reagents

The MAG-XTRACT RNA extraction kit from LGC Biotecnologia (Brazil), MagneSphere streptavidin paramagnetic particles (PMPs) from Promega (USA), and M-MuLV Reverse Transcriptase enzyme from New England Biolabs (USA) were obtained. Hexamer primers and Dithiothreitol (DTT) were sourced from Invitrogen (USA), RNAsin from Promega (USA), deoxyribonucleotide triphosphate (dNTPs) from Sinapse Inc. (USA), and the Taq Platinum DNA Polymerase Chain Reaction kit from Invitrogen (USA). The set of primers for PCR and LAMP reactions was synthesized by Alpha DNA (Canada), and the WarmStart Colorimetric LAMP 2X Master from New England Biolabs (USA) was employed in the LAMP reaction.

### Biological samples

The samples used in this study were provided in partnership with a clinical analysis laboratory in the city of Catalão, Goiás, Brazil. Participants who agreed to participate in this study signed an informed consent form. The Research Ethics Committee of the Federal University of Goiás approved this work, on 26/10/2016, with a number CAAE 59323816.9.0000.5083. For this investigation, we collected a total of 60 serum samples from volunteers, along with relevant demographic information, including age, sex, and the results of their serological tests, spanning the period from 2019 to 2022. Samples from individuals of all age groups were included in the study. As positive controls, tests with the four dengue serotypes (DENV-1, DENV-2, DENV-3, and DENV-4) were performed with plasmids containing the 5′ untranslated region (UTR) from the virus.

### RNA extractions with magnetic beads

**Fig 1:**
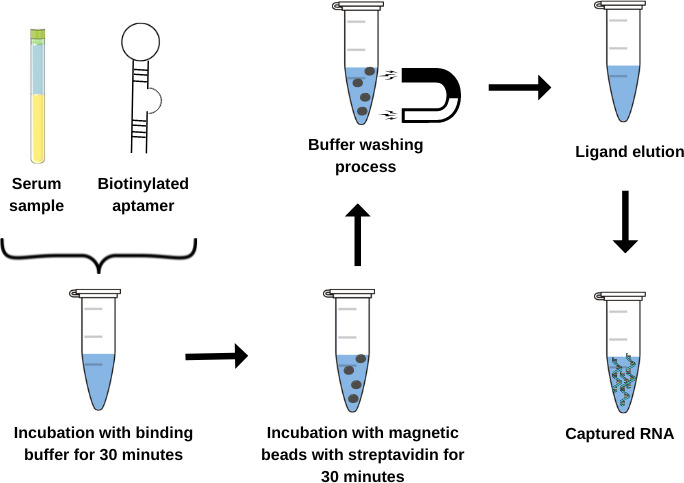
Workflow for the aptamer-based capture test. The biotinylated aptamer was directly incubated with human serum and then bound to paramagnetic particles, facilitating the capture of dengue viral RNA. Subsequent washing processes were conducted, and the genetic material of the virus underwent testing in the molecular assays outlined in the study.

Commercial kit and APTA-B07 capture test based on magnetic beads technology the RNA extracting was performed using 150 µL of serum of each participant of this study. The manufacturer’s protocol was followed, with washing, binding, and cleaning steps. Based on the previous aptamer and dengue capture method described by our research group (16, 21), a protocol for the genetic material of the dengue virus was performed. Using the APTA-B07 aptamer that binds to the 5′-UTR portion of the dengue virus (16), the RNA capture test was performed on the biological samples. For this method, just 1/3 (50 µL) of serum volume used in the MAG-XTRACT RNA extraction kit was incubated to the APTA-B07 biotinylated and paramagnetic particles complexed with streptavidin. After 1.5 hours, including the washing processes and with the aid of a magnetic platform, captured viral RNA was eluted in water treated with diethylpyrocarbonate (DEPC) (21). The workflow for the aptamer-based capture test is presented in Fig. 1.

### Reverse transcription and RT-PCR assay with RNAseP as quality control

The RT reaction was performed for the RNA samples extracted for both methods. The cDNA was obtained following the reaction with 5× buffer (50 mM Tris-HCl, 75 mM KCl, 3 mM MgCl2, and 10 mM DTT) 40 U of MMLV-RT, 10 mM of Dithiothreitol (DTT), 10 U of RNAsin, 0.2 mM of dNTPs, 5 µM of hexamer primers, 10 µL of the RNA, and water to complete the final volume of 20 µL. The reaction was incubated at 37°C for 1 hour. To validate the RNA extraction processes, a PCR was performed with RNAseP primers, which was used as an internal control. Primers are described according to Centers for Disease Control and Prevention guidelines for the detection of SARS-CoV-2 by RT-PCR (22). The reaction conditions were as follows: 1× reaction buffer (200 mM Tris-HCl, 500 mM KCl 500 mM), 2.5 U of Taq DNA Polymerase enzyme, 1.5 mM of MgCl2, 0.2 mM of dNTPs, 0.75 µM of each forward and reverse primer, 3 µL of cDNA and ultrapure water to complete the final volume of reaction to 25 µL. PCR cycling conditions were 95°C for 5 minutes followed by 40 cycles of 30 seconds at 55°C, and 60 seconds at 72°C. Results were visualized on a 1.5% agarose gel.

### RT-PCR assay for dengue detection

To detect the presence or absence of the virus in the selected samples, and for comparative analysis, an RT-PCR reaction was performed to detect the 5′-UTR untranslated portion, with primers according to Aquino et al. (23). The reaction conditions were 1× reaction buffer, 2U of Taq DNA Polymerase enzyme, 1.5 mM of MgCl2, 0.2 mM of dNTPs, 5 µM of each primer, 2 µL of cDNA, and ultrapure water to complete the reaction volume to 25 µL. PCR cycling conditions were 95°C for 5 minutes followed by 40 cycles of 30 seconds, 55°C for 45 seconds, 72°C for 2 minutes, and a final extension of 72°C for 10 minutes (21). Results were visualized on a 1.5% agarose gel. The primer sequences used to detect RNAseP and the 5′-UTR of the dengue virus are provided in Table 1. PCR was performed using the samples extracted by the commercial kit and PCR with the samples from the capture using APTA-B07.

**TABLE 1:** Primer sequences for RT-PCR and APTA-RT-PCR reactions

Target	Primer sequence (5’−3’)	Amplification size (bp)
RNAseP—Forward	5′- AGATTTGGACCTGCGAGCG-3′	65
RNAseP—Reverse	5′- GAGCGGCTGTCTCCACAAGT-3′	
5′-UTR DENV—Forward^*a*^	5′-AGTTGTTAGTCTACGTGGACCGA-3′	150
5′-UTR DENV—Reverse^*a*^	5′-CGCGTTTCAGCATATTGAAAG-3′	

^*a*^ Primers according to Aquino et al. (23).

### Bioinformatics analyses

The potential interactions between the APTA-B07 (16) together with the PCR sequences of the RNAseP (#NM_006413.5) and the 5′-UTR of DENV-1 (#OR418422.1) were performed using the RNAstructure web server (24). The bifold method was employed to predict intramolecular base pairs for two sets of sequences (APTA-B07 *versus* DENV-1, APTA-B07 *versus* RNAseP) and to determine the free energy associated with these structures.

### RT-LAMP assay for dengue detection

Using RT from biological samples where RNA was extracted by both procedures described above, tests were carried out using the WarmStart Colorimetric Lamp 2X Master Mix kit. The reaction mix, with a final volume of 15 µL was performed containing 1× MasterMix, 2.5 µL of cDNA, 2.2 µM (0.7: 0.1: 0.2) of the FIP and BIP, F3 and B3 and Loop primers. The primers were designed based on the virus RNA target. A set of genomes has been taken and aligned from the National Center for Biotechnology Information (NCBI) database (https://www.ncbi.nlm.nih.gov/), and the control genome for the designer was # OR418422.1 for DENV-1, #KP723479.1 for DENV-2, #JF808126.1 for DENV-3, and #KT276273.1 for DENV-4. The FIP, BIP, F3, B3, and loop sequence primers were obtained according to Notomi et al. (11). The Primer Explorer software (https://primerexplorer.jp/e/) assisted as a tool in the design of primers. The reaction was incubated at 65°C for 30 minutes, and the colorimetric detections were annotated as yellow for positive and pink for negative cases, according to the manufacturer’s instructions. To verify the specificity of the synthesized primers for the DENV, the RT-LAMP reaction was performed for each one of the serotypes (DENV-1, DENV-2, DENV-3, and DENV-4) cloned in plasmid transformed into *E. coli*, and with two biological samples positive for ZIKV and a synthetic sequence of 5′-UTR for this virus.

### Data analysis

Analysis for clinical parameters, including sensitivity, specificity, accuracy, positive predictive value (PPV), negative predictive value (NPV), Fisher’s exact test, and receiver operating characteristic curve (ROC), were conducted using MedCalc Statistical Software version 19.2.6 (MedCalc Software bv, Ostend, Belgium; https://www.medcalc.org; 2020). For ROC curve construction, the results were categorized as follows: excellent for area under the curve (AUC) values between 0.9 and 1, good for AUC values between 0.8 and 0.9, fair for AUC values between 0.7 and 0.8, poor for values of AUC between 0.6 and 0.7, and failed for AUC values between 0.5 and 0.6. Data analysis for research participants was conducted using Microsoft Excel (USA) software. For association analysis among molecular and serological tests, stratified by participants' gender and age in the research study, the statistical significance was considered when the p-value was less than or equal to 0.05.

## RESULTS

### APTA-RT-PCR has the same dengue virus detection sensitivity as RT-PCR

**Fig 2:**
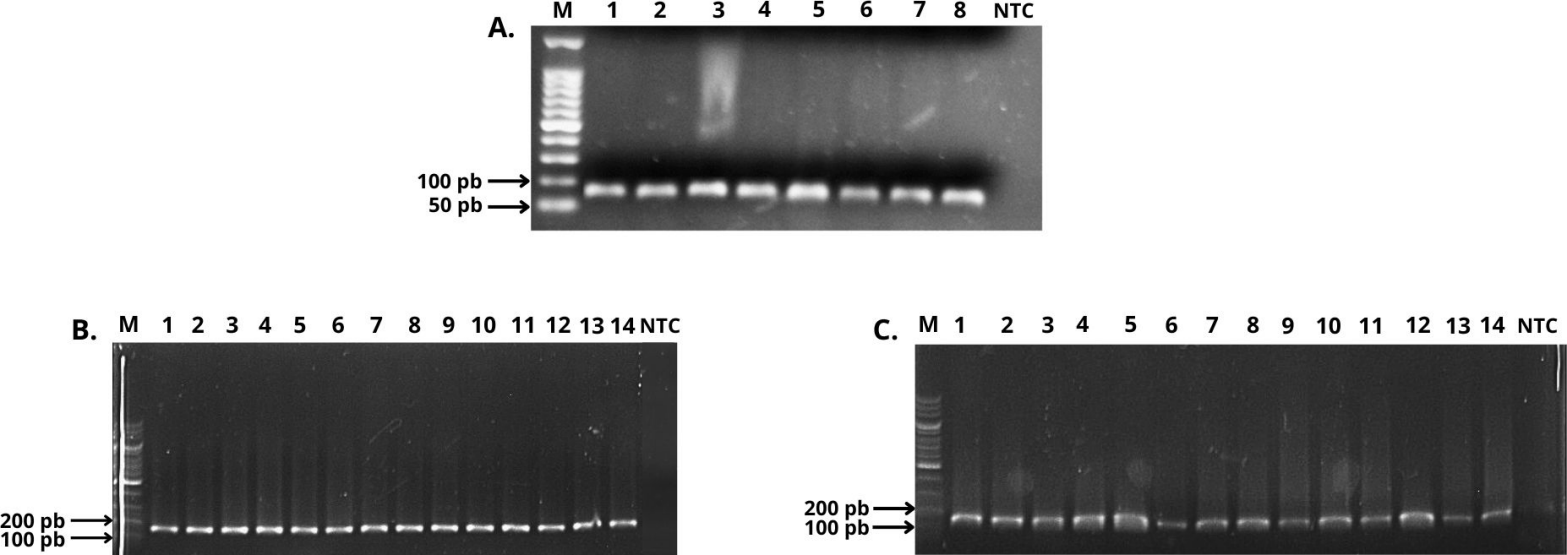
Electrophoresis gels for RT-PCR and APTA-RT-PCR for RNAseP and for 5′-UTR of DENV. (**A**) RT-PCR for RNAseP as quality control of RNA extraction of serum samples. Agarose gel electrophoresis shows DNA bands at the expected molecular size of about 65 bp, confirming the amplification of the RNAseP as constitutive control. Lanes 1–4: commercial kit extraction. Lanes 5–8: APTA-B07 capture test. M: 50 bp DNA ladder. NTC: no template control. (**B**) RT-PCR for detection of dengue virus. M: 100 bp DNA ladder. Lane 1: positive control. Lanes 2–14: DENV-positive samples with the expected molecular size of 150 bp. NTC: no template control. (**C**) APTA-RT-PCR for detection of dengue virus. M: 100 bp DNA ladder. Lane 1: positive control. Lanes 2–14: DENV-positive samples with the expected molecular size of 150 bp. NTC: no template control.

Aptameric capture of the viral RNA in combination with RT-PCR (APTA-RT-PCR) demonstrates comparable sensitivity to RT-PCR in detecting the dengue virus. The integrity of RNA extraction was validated using the RNAseP gene as a control. The commercial kit successfully extracted RNA, as indicated by the presence of a 65 bp product. Surprisingly, the aptamer-based capture method could also extract RNA from the RNAseP gene. Both procedures yielded sufficient RNA for target gene amplification, as shown in Fig. 2A. Figure 2B and C represents the results of RT-PCR tests conducted to detect the presence or absence of the virus in RNA extracted from biological samples, using the commercial and aptamer procedures, respectively. Consistent results were obtained, with the genetic material of the dengue virus detected in 67% (40 out of 60) of the cases by RT-PCR. The selected 5′-UTR region, known for its conservation across serotypes, enabled the detection of any serotypes present in the samples, thus justifying its use for diagnostic purposes.

**Fig 3:**
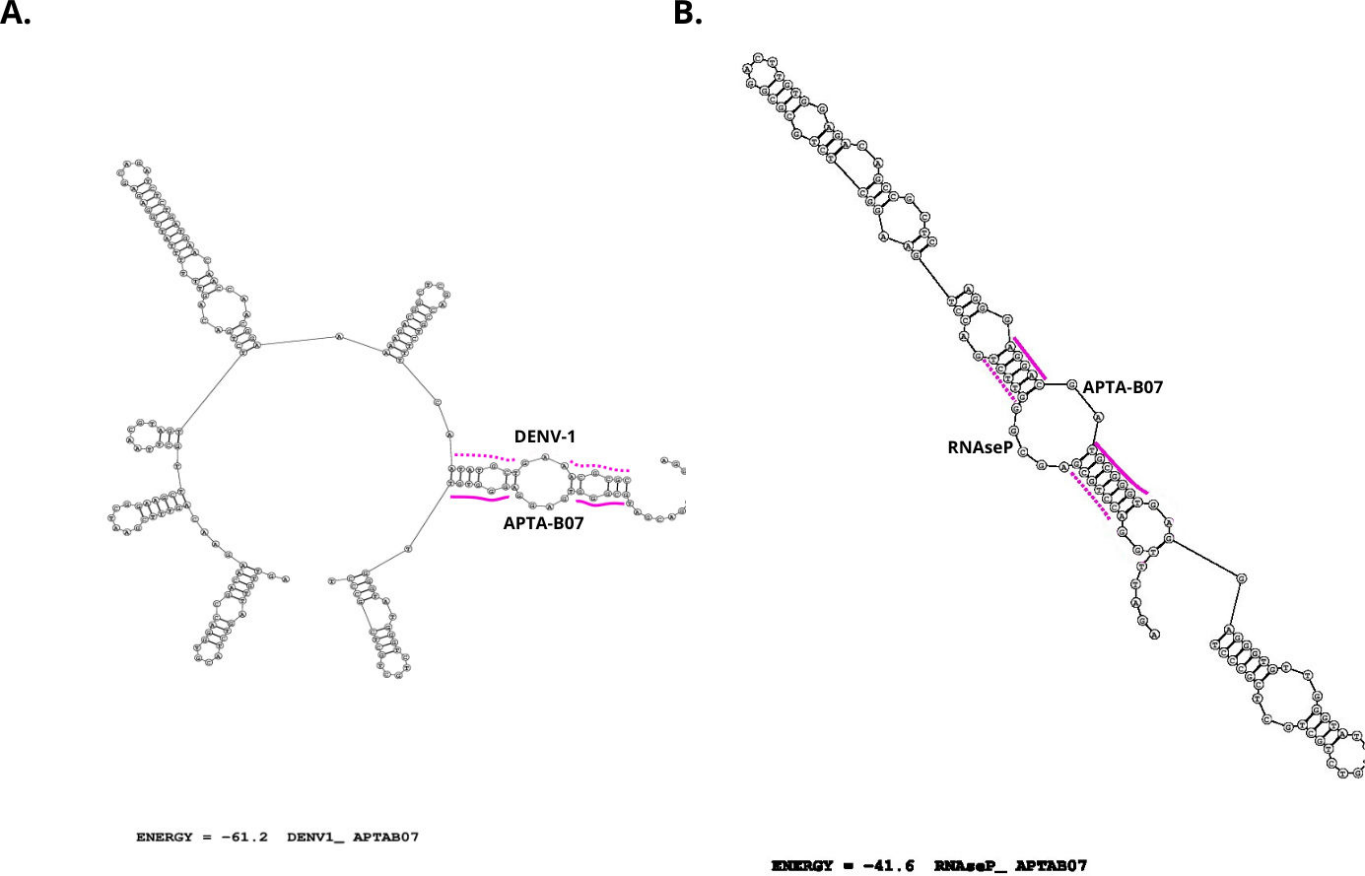
Intramolecular base pairs for APTA-B07 *versus* DENV-1 and APTA-B07 *versus* RNAseP. (**A**) Predicted structure between APTA-B07 with 5′-UTR from DENV-1. (**B**) Predicted structure between the human RNAseP and APTA-B07. The free energies for APTA-B07 *versus* DENV-1 and DENV-1 and APTA-B07 structures were −61.2 and −41.6, respectively.

The APTA-RT-PCR bands observed in Fig. 2A (lanes 5–8) demonstrate the efficacy of aptamer APTA-B07 in capturing RNAseP transcripts. Complementary to this, bioinformatic analyses reveal possible interactions between the aptamer and DENV1, the target of SELEX, as well as between APTA-B07 and RNAseP (Fig. 3A and B, respectively). The free energies for the APTA-B07 versus DENV-1 and APTA-B07 versus RNAseP structures were −61.2 and −41.6, respectively, indicating a more stable interaction between APTA-B07 and the target of the SELEX, the 5′-UTR of the dengue virus.

### RT-LAMP and APTA-RT-LAMP are effective in detecting the dengue virus

**Fig 4:**
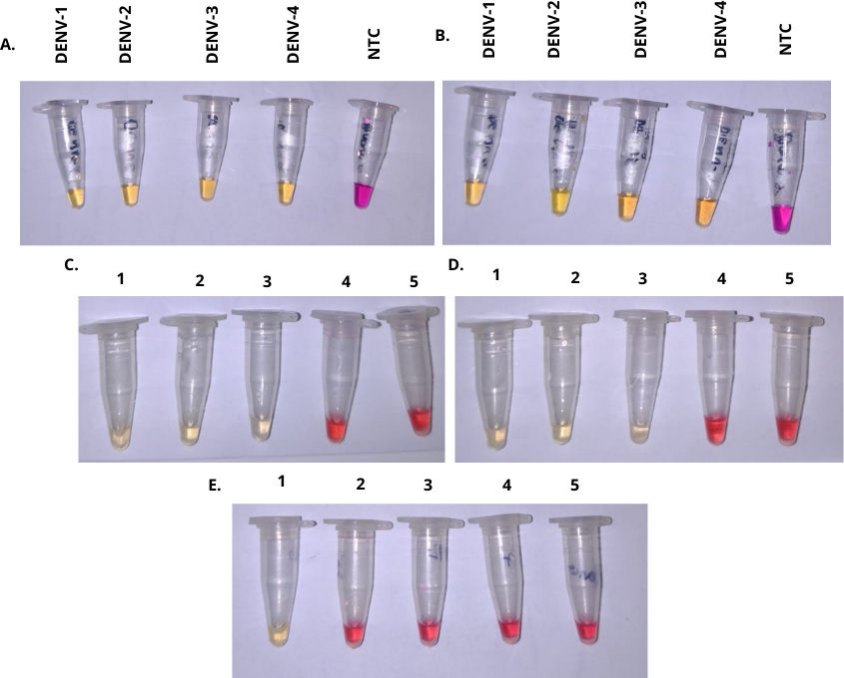
Colorimetric RT-LAMP and APTA-RT-LAMP assays for detection of dengue virus. (**A**) RT-LAMP assay with plasmids of dengue serotypes. NTC: no template control. (**B**) APTA-RT-LAMP assay with plasmids of dengue serotypes. NTC: no template control. (**C**) RT-LAMP: (1) Positive DENV control (2). Positive biological sample (3). Positive biological sample (4). Negative biological sample (5). No template control. (**D**) APTA-RT-LAMP: (1) Positive DENV control (2). Positive biological sample (3). Positive biological sample (4). Negative biological sample (5). No template control. (**E**) LAMP specificity assay: (1) DENV positive sample (2). Synthetic ZIKV sample (3). Positive ZIKV biological sample (4). Positive ZIKV biological sample (5). No template control.

Based on the results shown in Fig. 4, a noticeable color change is observed between positive and negative results. Both RT-LAMP and APTA-RT-LAMP methodologies successfully amplified all four DENV serotypes, as shown in Fig. 4A and B, respectively. Furthermore, both methods effectively detected the presence of the dengue virus in biological samples, as illustrated in Fig. 4C and D. Figure 4E demonstrates that amplification did not occur in reactions containing synthetic or biological samples of zika virus genetic material, while amplification was only observed in the reaction containing a dengue sample.

Tables 2 to 4 illustrate that APTA-RT-LAMP yielded results closely aligned with APTA-RT-PCR and RT-PCR, established as our gold standard for result comparisons. In the RT-LAMP results, 53% (32 out of 60) of the samples tested positive for dengue detection, while 47% (28 out of 60) were negative when compared with RT-PCR (Table 2). Incorporating aptamer together with the LAMP as a process for dengue detection, increased the test positivity from 53% to 63% (38 out of 60) (Table 3). The APTA-RT-PCR results mirrored the RT-PCR data, with 40 detected and 20 not detected (Table 4).

**TABLE 2:** Comparison between RT-LAMP and RT-PCR results

RT-LAMP	RT-PCR		
	Detected	Non-detected	Total
Detected	32	0	32
Non-detected	8	20	28
**Total**	40	20	60

**TABLE 3:** Comparison between APTA-RT-LAMP and RT-PCR results

APTA-RT-LAMP	RT-PCR		
	Detected	Non-detected	Total
Detected	38	0	38
Non-detected	2	20	22
Total	40	20	60

**TABLE 4:** Comparison between APTA-RT-LAMP and APTA-RT-PCR results

APTA-RT-LAMP	APTA-RT-PCR		
	Detected	Non-detected	Total
Detected	38	0	38
Non-detected	2	20	22
Total	40	20	60

Table 5 presents the diagnostic parameters of RT-LAMP and APTA-RT-LAMP compared with RT-PCR. The data indicate an accuracy of 98% for APTA-RT-LAMP, which makes it a potential gold standard technique for detecting DENV genetic material, similar to conventional RT-PCR.

**TABLE 5:** RT-LAMP and APTA-RT-LAMP diagnostic performance indexes

	% Sensitivity (95% CI)	% Specificity (95% CI)	% Accuracy (95% CI)	% PPV (95% CI)	% NPV (95% CI)
RT-LAMP	80 (64.35 to 90.95)	100 (83.16 to 100)	91 (80.76 to 96.85)	100 (89.11 to 100)	71.4 (57.36 to 82,29)
APTA-RT-LAMP	95 (83.08 to 99.39)	100 (83.16 to 100)	98 (90.12 to 99.87)	100 (90.75 to 100)	90.91 (72.15 to 97.48)

^*a*^ PPV, positive predictive value; NPV, negative predictive value.

**Fig 5:**
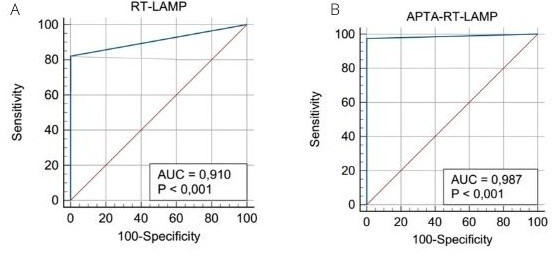
ROC curve of RT-LAMP and APTA-RT-LAMP. (**A**) RT-LAMP and (**B**) APTA-RT-LAMP. The diagnostic accuracy of both tests was notably high, with area under the curve (AUC) values exceeding 0.9. Importantly, the APTA-RT-LAMP test showed superior performance compared to the RT-LAMP, achieving a higher AUC value approaching the ideal 1.

Both tests exhibited an area under the curve (AUC) greater than 0.9, as demonstrated in Fig. 5. This result is considered excellent. However, the APTA-RT-LAMP test achieved a higher AUC value closer to 1, which represents an ideal test score. The use of these indices facilitates the understanding of the strengths and weaknesses of the tests proposed in this study. The seroprevalence analysis within the cohort revealed that 45% (27 out of 60) exhibited dual positivity for Immunoglobulin M (IgM) and Immunoglobulin G (IgG), 17% (10 out of 60) were exclusively positive for IgM, and 8% (5 out of 60) were exclusively positive for IgG. In addition, 10% of cases (two for IgG and four for IgM) exhibited cross-reactivity, manifesting seroprevalence, while the RT-PCR assay did not detect the dengue virus. Concerning the demographic profile of the investigated cohort, a predominance of detected cases was observed among males, particularly within the age bracket of 18–30 years, as outlined in Table 6.

**TABLE 6:** Distribution of dengue cases based on RT-LAMP, APTA-RT-LAMP, and serological tests, stratified by participants' gender and age in the research study

Patient data	RT-LAMP		APTA-RT-LAMP		Serological test	
	Detected	Non-detected	Detected	Non-detected	Reactive	Non-reactive
Sex						
Male	17/32	20/28	21/38	16/22	28/42	9/18
Female	15/32	8/28	17/38	6/22	14/42	9/18
Age group						
<18–30 years	14/24	10/24	16/24	8/24	16/24	8/24
31–50 years	8/22	14/22	10/22	12/22	15/22	7/22
>51 years	10/14	4/14	11/14	3/14	13/14	1/14
Serological test^*a*^						
Positive	31/44	13/44	37/44	7/44	N/A	N/A
Negative	1/16	15/16	1/16	15/16	N/A	N/A

^*a*^ Fisher’s exact test for RT-LAMP versus serological tests presented *P* < 0.000012, and for APTA-RT-LAMP versus serological tests, the *P* value was <0.000001.

In general, molecular or serological data were not associated with demographic data stratified by sex and age. On the other hand, a positive association was found between detection by RT-LAMP or APTA-RT-PCR with seropositivity by IgM and/or IgG (<0.000012 and <0.000001, respectively) (Table 6).

## DISCUSSION

Dengue is a disease of significant importance to public health in tropical and subtropical countries. In our study, we employed RT-LAMP for the detection of the dengue virus in serum samples, using two methods for obtaining viral genetic material. The methods included a conventional RNA extraction protocol using a commercial kit, which was contrasted with a protocol utilizing the previous capture method as described by our research group (16, 21), involving an aptamer with an affinity to the dengue virus. Consequently, in conjunction with the RT-LAMP, we developed an APTA-RT-LAMP. Both methods demonstrated a straightforward way to execute these assays and a naked-eye observation of the color developing after the reaction, allowing the virus detection. The use of the aptamer improved sensitivity rates, producing results close to those of RT-PCR.

Regarding the temporal investment in each assay, the execution of the RT-PCR technique, from obtaining viral RNA using a commercial kit to visualizing the results through gel electrophoresis, takes approximately 7 hours. In the case of APTA-RT-PCR, approximately 6 hours are required for completion. For RT-LAMP, the time interval from RNA extraction to result in visualization is around four hours with 30 minutes for the color change indicating the presence or absence of the dengue virus. For APTA-RT-LAMP, the analogous process takes about three and a half hours. One distinctive advantage of LAMP methods is the absence of sample manipulation for result visualization, as the observation is made with the naked eye.

The LAMP technique has gained attention from researchers and diagnostic laboratories due to its reduced analysis time (25, 26). It has been successfully applied in the detection of various pathogens. The ongoing SARS-CoV-2 pandemic since 2020 has further spurred research and utilization of molecular diagnostics based on RT-LAMP, where reverse transcription also takes place given the viral nature of SARS-CoV-2 (27).

An example of the application of LAMP is the work by Becherer et al. (28), where LAMP was used for the simultaneous detection of the bacteria *Treponema pallidum* and *Haemophilus ducreyi*, which cause sexually transmitted infections (STIs). This demonstrates the versatility of the LAMP technique in detecting diverse pathogens. The key difference between RT-LAMP and APTA-RT-LAMP is the incorporation of aptamers in the latter, which simplifies and reduces the cost of the RNA extraction process compared to commercial kits. Aptamers have gained attention in the field of diagnosis in recent years due to their specificity and compatibility with other technologies (29).

In 2016, the World Health Organization (WHO) endorsed the adoption of the Loopamp MTBC Detection kit, developed by the Japanese company Eiken Chemical Company, for tuberculosis detection in select African and Asian nations. The recommendation involved incorporating this test either as an alternative or as a supplementary diagnostic alongside the sputum smear test (30).

Ahn et al. (31) showcased the integration of RT-LAMP with gold nanoparticles for the colorimetric detection of the Japanese encephalitis virus (JEV), a flavivirus. Siriyasatien et al. (32) employed colorimetric RT-LAMP to identify the Asian lineage zika virus in mosquitoes, facilitating the monitoring of infected mosquitoes. In a similar vein, Calvert et al. (33) successfully detected the zika virus in serum and urine using the naked eye with colorimetric RT-LAMP. Baek et al. (34) utilized the RT-LAMP colorimetric detection kit to identify the Thrombocytopenia Syndrome Virus (SFTSV), whose infection in humans leads to symptoms resembling dengue. Biswas et al. (35) utilized a paper microfluidic platform for the detection of the dengue virus through RT-LAMP. These studies collectively underscore the promising potential of the LAMP technique for point-of-care platforms for diverse viral targets.

Ma, Chen, and Lee (36), who coupled an aptamer specific to the H1N1 virus with magnetic beads for virus capture, demonstrated the use of aptamers for viral target selection within a sample. The detection was then performed using RT-LAMP on a microfluidic chip, showcasing the potential of combining these two molecular biology technologies to enhance and facilitate diagnosis.

Building on these findings, our capture test methodology was inspired by the work of Silva et al. (21) that employed APTA-RT-PCR for the detection of Zika virus (ZIKV) and dengue virus (DENV). While they used serum samples, we employed APTA-RT-LAMP for dengue virus detection, enabling visual observation of results with the naked eye and utilizing simpler equipment such as a dry bath, which reduces testing costs.

The ROC curve results obtained in our study were excellent, with an AUC exceeding 0.9 for all molecular tests performed. This indicates a high level of performance for both RT-LAMP and APTA-RT-LAMP compared to RT-PCR, without any false-positive results.

One of the challenges with serological diagnoses is the occurrence of false positives, particularly due to cross-reactions among viruses within the same family, such as dengue and Zika. Despite being commonly used due to their lower cost, serological tests have limitations (37). However, the use of aptamers improved the sensitivity of our test by specifically capturing the genetic material of the dengue virus, thus reducing the risk of false positives and improving molecular cost-efficiency by eliminating the need for RNA extraction.

Another advantage of the aptamer capture method is the avoidance of low-temperature centrifugation, allowing its use in less-equipped laboratories. In addition, the process optimizes time, taking less than 2 hours to obtain viral RNA (21).

The APTA-RT-PCR results revealed a positive detection of the constitutive gene RNAseP using our aptamer, as corroborated by homology analysis. The data indicate the potential use of aptamers to retain RNAseP transcripts as a reliable host constitutive control in APTA-Loop-mediated Isothermal Amplification (LAMP) assays. Although there is literature using aptamers for the isolation of RNAseP (38, 39), no report was found using aptamers to capture constitutive genes in APTA-RT-PCR or APTA-RT-LAMP assays. This highlights a potential avenue for further exploration in the development of aptamer-based methods for molecular diagnostics.

The colorimetric LAMP method we employed offers further advantages by eliminating the need for additional sample manipulation steps during reaction detection, reducing the risk of contamination and the requirement for sophisticated equipment (25).

Given the similarities between zika and the dengue virus, interpreting serological tests accurately becomes challenging. Amplification-based methods targeting genetic material offer greater specificity and are less prone to false positives (40). In our study, the LAMP reactions did not amplify the zika virus genetic material in the samples that were confirmed to be negative by RT-PCR, serving as a specificity control. This confirms that our assay does not exhibit cross-reactivity with the genetic material of the zika virus.

Dengue remains a significant public health problem, particularly in the Americas. According to data from the Pan American Health Organization. There has been a significant increase in the number of dengue cases in the past decade, with millions of cases registered. Due to the limitations of current detection methods and the underreporting of the disease, it is crucial to search for simpler diagnostic approaches that provide reliable results (41).

The combination of the LAMP method with aptamers has yielded favorable results in our study. However, further research with a larger sample size and extensive validation tests will be necessary. Nevertheless, our preliminary results demonstrate the potential of utilizing these two molecular technologies to develop a robust diagnostic tool for the detection of the dengue virus. Once standardized, this approach can also be applied to other neglected tropical diseases, opening new possibilities for improved diagnosis and surveillance.
